# Convolutional neural network applied to preoperative venous-phase CT images predicts risk category in patients with gastric gastrointestinal stromal tumors

**DOI:** 10.1186/s12885-024-11962-y

**Published:** 2024-03-01

**Authors:** Jian Wang, Meihua Shao, Hongjie Hu, Wenbo Xiao, Guohua Cheng, Guangzhao Yang, Hongli Ji, Susu Yu, Jie Wan, Zongyu Xie, Maosheng Xu

**Affiliations:** 1 Department of Radiology, Tongde Hospital of Zhejiang Province, Hangzhou, Zhejiang, China; 2 Department of radiology, The First Affiliated Hospital of Zhejiang Chinese Medical University (Zhejiang Provincial Hospital of Chinese Medicine), Hangzhou, Zhejiang China; 3 Department of Radiology, The Sir Run Shaw Hospital, Zhejiang University School of Medicine, Hangzhou, Zhejiang China; 4 Department of radiology,The First Affiliated Hospital, Zhejiang University School of Medicine, Hangzhou, Zhejiang China; 5 Jianpei Technology, Hangzhou, Zhejiang China; 6 Department of Radiology, The First Affliated Hospital of Bengbu Medical University, Bengbu, Anhui China

**Keywords:** Computed tomography, Gastric stromal tumors, Convolutional neural network

## Abstract

**Objective:**

The risk category of gastric gastrointestinal stromal tumors (GISTs) are closely related to the surgical method, the scope of resection, and the need for preoperative chemotherapy. We aimed to develop and validate convolutional neural network (CNN) models based on preoperative venous-phase CT images to predict the risk category of gastric GISTs.

**Method:**

A total of 425 patients pathologically diagnosed with gastric GISTs at the authors’ medical centers between January 2012 and July 2021 were split into a training set (154, 84, and 59 with very low/low, intermediate, and high-risk, respectively) and a validation set (67, 35, and 26, respectively). Three CNN models were constructed by obtaining the upper and lower 1, 4, and 7 layers of the maximum tumour mask slice based on venous-phase CT Images and models of CNN_layer3, CNN_layer9, and CNN_layer15 established, respectively. The area under the receiver operating characteristics curve (AUROC) and the Obuchowski index were calculated to compare the diagnostic performance of the CNN models.

**Results:**

In the validation set, CNN_layer3, CNN_layer9, and CNN_layer15 had AUROCs of 0.89, 0.90, and 0.90, respectively, for low-risk gastric GISTs; 0.82, 0.83, and 0.83 for intermediate-risk gastric GISTs; and 0.86, 0.86, and 0.85 for high-risk gastric GISTs. In the validation dataset, CNN_layer3 (Obuchowski index, 0.871) provided similar performance than CNN_layer9 and CNN_layer15 (Obuchowski index, 0.875 and 0.873, respectively) in prediction of the gastric GIST risk category (All *P* >.05).

**Conclusions:**

The CNN based on preoperative venous-phase CT images showed good performance for predicting the risk category of gastric GISTs.

## Introduction

Gastric gastrointestinal stromal tumors (GISTs) account for 60–65% of all GISTs, followed by GIST_S_ of the small intestine (25–30%) and colorectal region (5%). GIST_S_ derive from interstitial cells of Cajal (ICC), and have a potential for malignancy [1, 2]. Independent prognostic factors for GISTs based on the National Institutes of Health (NIH) risk category criteria include tumor size and site, mitotic count, and tumor rupture [3]. Risk stratification is essential to identify and better define those patients with GISTs who are most likely to benefit from adjuvant imatinib therapy [4]. High-risk GISTs are considered to require a multidisciplinary approach to improve the prognostic outcome, such as one including adjuvant therapy and surgery [5, 6]. Therefore, it would be helpful to determine a precise preoperative risk rating to ensure appropriate adjuvant therapy and treatment for individual patients.

Abdominal contrast-enhanced computed tomography (CT) is the most commonly applied method for determining the signs of GISTs, such as calcification, hemorrhage, growth pattern, degree of enhancement, necrosis, and lymph node involvement [7, 8]. However, the resulting subjective interpretations have inevitable limitations because of differences in reader experience and understading in the definitions of imaging features, thereby motivating researchers to seek more objective and reliable predictive approaches [9].

Recently, the convolutional neural network (CNN) has become the typical algorithm for deep learning, and they are now widely used in the fields of diagnostic imaging, classification, and prediction in various diseases, including gastric cancer, breast cancer, and lung cancer [10–12]. With advantages in accuracy, objectivity, and reproducibility, CNN models applied to imaging data can discern important predictive features that may not be detected by the naked eye [13, 14]. Although several CNN image data models have been applied to endoscopic ultrasonography (EUS) imaging of gastrointestinal diseases, there is still a lack of research on their application to contrast-enhanced CT images of gastric GISTs [11, 15, 16]. We considered whether a CNN-based model applied to venous phase contrast-enhanced CT would be able to predict the risk rating of gastric GISTs, and adopted a newly developed CNN called Efficient Net to build and validate predictive models for this purpose [17].

## Materials and methods

### Training and validation datasets

**Fig. 1:**
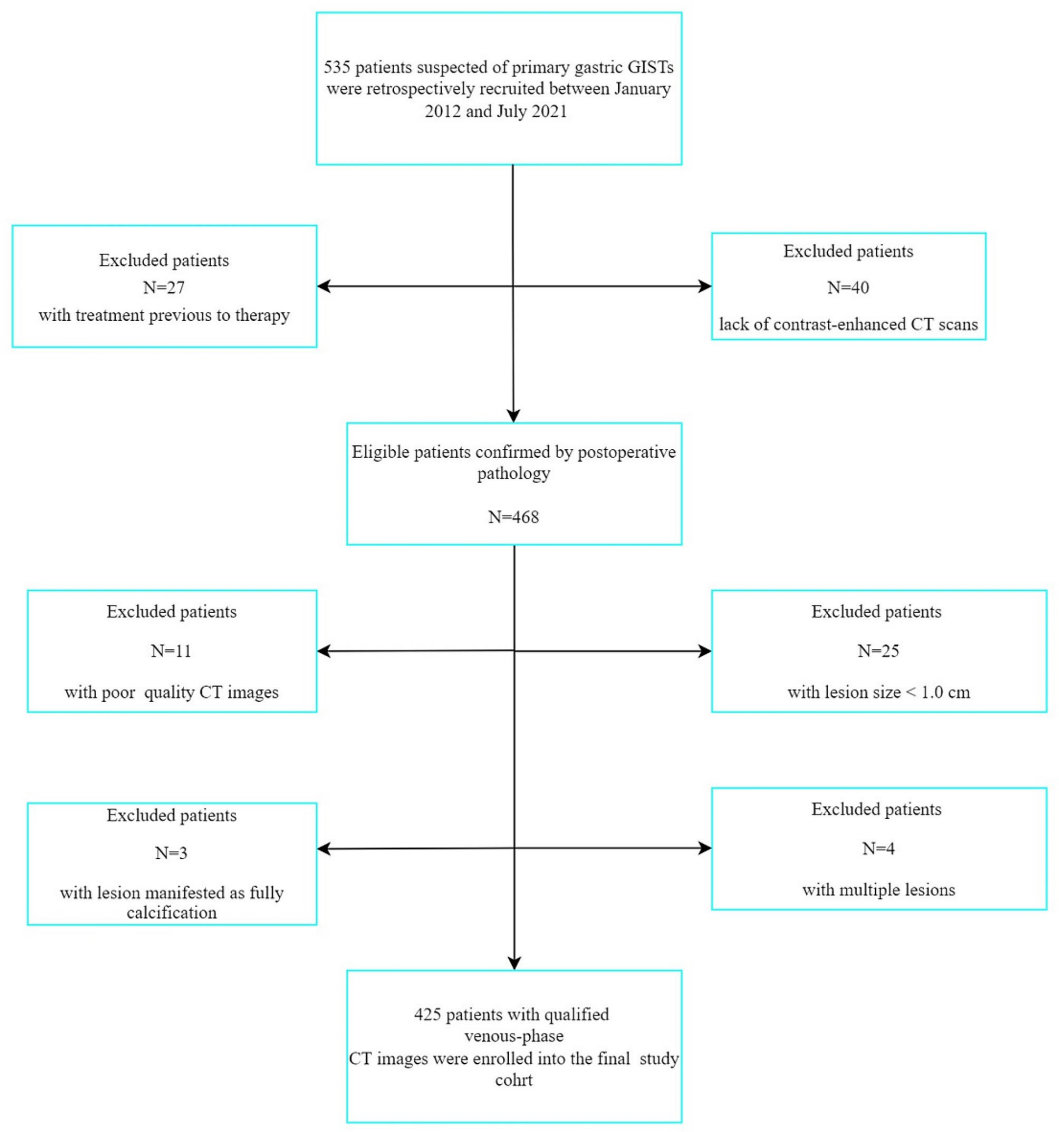
Flow chart of patient inclusion and exclusion. GISTs = gastrointestinal stromal tumors

This retrospective study was approved by Ethics Committee of Tongde Hospital of Zhejiang Province (Approval No. 2022-040) and waived the need of informed consent under ethical approval and consent to participant section under declaration section. Venous-phase contrast-enhanced CT images acquired between January 2012 and July 2021 were retrospectively analyzed from 4 four centers. Initially, 535 patients clinically suspected to have primary gastric GISTs were identified. The inclusion criteria were: (1) postoperative histopathological confirmation of gastric GIST; (2) contrast-enhanced CT acquired within 4 weeks before resection; (3) complete clinicopathologic materials; and (4) no chemotherapy previous to operation. The exclusion criteria consisted of poor CT image quality or lesion size < 1.0 cm (which may influence the segmentation of the target lesion), multiple lesions, and lesion manifesting as fully calcified. Details of the inclusion and exclusion criteria are demonstrated in Fig. 1. After applying these criteria, a total of 425 patients with pathologically diagnosed gastric GISTs were classified into either a training dataset (154, 84, and 59 patients with very low/low, intermediate, and high-risk, respectively) or a validation dataset (67, 35, and 26 patients with very low/low, intermediate, and high-risk, respectively). Clinical data including sex, mean age, and symptoms (hematemesis and/or melena) were also collected.

### CT examinations

All patients in the training and validation groups underwent contrast-enhanced CT examinations on one of the following CT scanners: SOMATOM Emotion16/64, Definition AS/Dual Source (SIEMENS Healthineers), and Optima CT680 (GE). All subjects were required to intake 500–1000 ml of water over 15 min before the CT scanning and to have fasted for at least 4 h. Arterial phase and portal venous phase images were acquired with delays of 25–30 s and 50–70 s after injection, respectively. All CT imaging was acquired using a tube voltage of 120–130 kV, tube current of 200–300 mA, slice thickness of 1.5–5.0 mm, and an intravenous injection of 80–120 ml of contrast medium delivered using an injection rate of 3–4 ml/s according to the patient’s weight (1.0 ~ 1.5 ml/kg). The following quantitative CT features of gastric GISTs were analyzed: mean CT value of unenhanced image (CT_U_), mean CT value of arterial phase (CT_A_), mean CT value of portal venous phase (CT_V_), longest dimension (LD), and shortest dimension (SD). The enhancement degree in the arterial phase and portal venous phase (DEAP and DEPP) referred to the results of CT_A_ and CT_V_ minus CT_U_, respectively. Qualitative CT features recorded included location, contour, growth pattern (endophytic, exophytic, and mixed), necrosis, calcification, surface ulceration, lymph node involvement (LN), hemorrhage, intratumoral vessel, peritumoral exudation, and necrosis under the tumor wall. Necrosis was defined according to an unenhanced CT value from − 20 HU to 20 HU, and the presence of calcification as a CT value above 100 HU. Surface ulceration was defined as the endoluminal surface of the lesion displaying localized tissue loss [18].The longest dimension and shortest dimension of the lesion were measured on axial images. The CT image analysis was retrospectively completed by two experienced radiologists (JW and MHS) with 17 years and 5 years who were blinded to the clinical details of the patients, and any inconsistencies were solved by consensus.

### Image preprocessing and CNN model

**Fig. 2:**
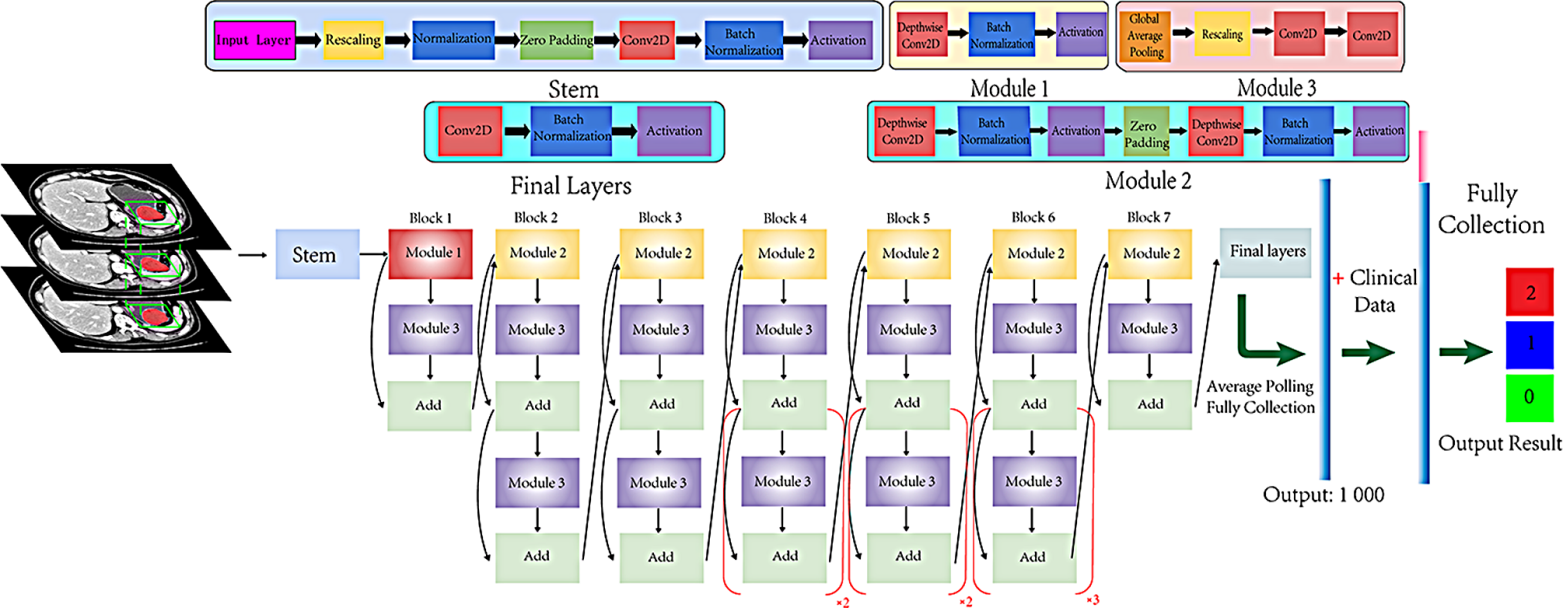
Proposed convolutional neural network (CNN) workflow for gastric GISTs risk rating

The venous-phase CT images were exported to ITK-SNAP software (open source, www.itk-snap.org) for manual segmentation, which was accomplished by the two experts who were blinded to the gastric GIST risk ratings when performing the segmentation. All the venous-phase CT images were performed with the homogenization process, including (1) data integration, (2) data washing (hiding patient information), (3) data standardization (denoising, unifying window width and window level), (4) data normalization, and (5) data label after structuring. The slice thickness of the venous-phase CT images was interpolated to 2 mm and the image slice with the largest lesion was determined according to the labeled tumor mask slice. Subsequently, an array of 512 × 512 × 3 was obtained from the upper and lower 1, 4, and 7 layers of the maximum mask slice, which form the models of CNN_layer3, CNN_layer9, and CNN_layer15, respectively. Then, the window width and window position were set to [40, 300]. Data normalization was performed by mapping the values to the range [-1, 1], and the images were then input into the network. The EfficientNet_b1, comprising of a stem, seven blocks, average polling, and full connection, was trained on the image data to establish the predictive model. The key procedures of the stem block consisted of convolution and batch normalization. The main operations of the seven blocks involved four steps: (1) convolution; (2) subsampling; (3) batch normalization; and (4) bouncing connection. A vector of length 1000, which was extracted by efficientnet-b1 from venous-phase CT images, was converted to a vector of 1023 after collecting 23 fields of clinical material. Finally, a vector of length three including low/low, intermediate, and high-risk three risk categories was output after the fully connected layer, and the probability of three categories were output using a softmax function. The detailed structure is shown in Fig. 2.

### Statistical analysis

The statistical analyses were performed using Python version 3.6 (Python Software Foundation). The prediction performance of the models, including sensitivity, specificity, true positives (TP), false negatives (FN), and areas under the receiver operating characteristic curves (AUROCs), were assessed for both the training and validation datasets using standard definitions. Along with the above indicators, the macro-average receiver operating characteristic (ROC) and micro-average roc were also computed for each class, allowing all classifications to be treated equally by individually calculating the index of each classification and then taking the average of the results. The Obuchowski index was calculated to compare the ROC curves of between these CNN models.

Continuous variables are reported as mean ± standard deviation. Differences in continuous variables between test and training sets, and among the three risk groups, were analyzed using independent samples t tests and analysis of variance, respectively. Differences in categorical data between the test and training sets and among the three risk groups were analyzed using the χ2 test. All tests were unpaired, and a two-tailed P value of < 0.05 was considered statistically significant.

## Results

### Patient demographics and CT features in all cohorts

The clinical characteristics and CT features of our six datasets are summarized and compared in Table 1. There was no significant difference in sex, mean age, symptom, risk category, location, calcification, CT_U_, CT_A_, CT_V_, DEAP, or DEPP between the very low/low-risk, intermediate-risk, and high-risk groups in either the training dataset or validation dataset (all *P* >.05). The contour, growth pattern, necrosis, surface ulceration, LN involvement, hemorrhage, intratumoral vessel, peritumoral exudation, necrosis under the tumor wall, LD, SD, and LD/SD showed significant differences between the low-risk, intermediate-risk, and high-risk groups in both the training and validation datasets (all *P* <.05). Tumor location distribution between the training dataset and validation dataset was significantly different using independent sample T test analysis (with *P* <.05).

**Table 1:** Clinical characteristics and CT features of 425 patients with GSTs in both training and validation

	GSTt0 (*n* = 154)	GSTt1 (*n* = 84)	GSTt2 (*n* = 59)	*P*	GSTv0 (*n* = 67)	GSTv1 (*n* = 35)	GSTv2 (*n* = 26)	*P*	Pt&v
Sex (Male/Female)	78/76	38/46	31/28	.634^a^	38/29	16/19	18/8	.186^a^	.201^a^
Mean age(y)	60.85 ± 0.82	59.31 ± 1.50	58.85 ± 2.13	.399^B^	60.97 ± 1.61	62.77 ± 3.09	59.04 ± 4.16	.377^B^	.362^b^
Symptom				.075^a^				.191^a^	.642^a^
0	51	36	16		24	14	6		
1	86	35	30		33	17	11		
2	17	13	13		10	4	9		
Risk category									.980^a^
0	154				67				
1		84				35			
2			59				26		
Location*†				0.967				0.458	0.043
Cardia	6	3	3		3	2	1		
Fundus	51	27	16		18	6	3		
Body	86	48	37		35	24	19		
Antrum	11	6	3		11	3	3		
Contour*				<0.001				<0.001	0.726
Round	66	14	1		35	3	1		
Oval	59	14	8		23	7	1		
Irregular	29	56	50		9	25	24		
Growth pattern*				<0.001				<0.001	0.934
Endophytic	77	25	8		34	9	2		
Exophytic	56	44	22		25	20	9		
Mixed	21	15	29		8	6	15		
Necrosis*	49	65	51	<0.001	24	26	22	<0.001	0.779
Calcification	22	15	14	0.258	6	9	4	0.087	0.553
Surface ulceration*	14	24	38	<0.001	3	10	15	<0.001	0.937
LN*	4	5	10	0.002	0	2	4	0.005	0.492
Hemorrhage*	0	2	8	<0.001	0	0	2	0.018	0.721
Intratumoral vessel*	12	26	26	<0.001	10	10	16	<0.001	0.143
Peritumoral exudation*	0	2	9	<0.001	0	1	3	0.006	0.992
Necrosis under the tumor wall*	34	45	45	<0.001	14	20	17	<0.001	0.714
CT_U_ (HU)	35.32 ± 0.35	35.79 ± 0.64	34.33 ± 0.92	0.499	34.66 ± 0.95	35.81 ± 1.81	34.25 ± 2.44	0.706	0.642
CT_A_ (HU)	56.93 ± 1.64	59.63 ± 3.00	57.09 ± 4.28	0.430	57.74 ± 4.18	59.36 ± 8.00	56.53 ± 10.77	0.798	0.899
CT_V_ (HU)	69.05 ± 1.81	73.32 ± 3.32	68.92 ± 4.73	0.134	71.55 ± 6.17	74.73 ± 11.81	71.26 ± 15.89	0.717	0.258
DEAP (HU)	21.61 ± 1.44	23.84 ± 2.63	22.76 ± 3.75	0.534	23.09 ± 4.08	23.55 ± 7.81	22.28 ± 10.51	0.956	0.718
DEPP (HU)	33.73 ± 1.87	37.53 ± 3.42	34.59 ± 4.87	0.249	36.90 ± 6.56	38.92 ± 12.56	37.01 ± 16.90	0.890	0.193
LD (mm)*	27.43 ± 4.37	52.29 ± 8.01	88.85 ± 11.41	<0.001	26.40 ± 9.19	55.00 ± 17.60	74.88 ± 23.69	<0.001	0.470
SD (mm)*	22.38 ± 2.03	40.71 ± 3.71	62.72 ± 5.29	<0.001	21.66 ± 4.20	45.63 ± 8.04	56.49 ± 10.82	<0.001	0.905
LD/SD*	1.24 ± 0.001	1.28 ± 0.001	1.44 ± 0.001	<0.001	1.24 ± 0.001	1.24 ± 0.002	1.29 ± 0.001	0.010	0.167

Note.—Except where indicated, data are numbers of tumors. GST = Gastric stromal tumor. GSTt0/1/2 and /GSTv0/1/2 = training or validation data for risk 0/1/2 GS. y = years. M ± SD = mean ± standard deviation. Calculated with χ^2^ test (^a^), Analysis of variance (^B^) and independent sample T test (^b^). Symptom 0/1/2 = asymptomatic/symptoms without hematemesis, melena/ hematemesis and/or melena. Risk 0/1/2 = very-low and low/ intermediate/high risk groups. LN = Lymph node. CT_U_/CT_A_/CT_V_ = the CT attenuation value of unenhanced /arterial/venous phase. DEAP/DEPP = CT_A_ - CT_U_/ CT_V_ - CT_U_. LD = long dimension. SD = short dimension. Analysis of variance (*P*) and independent sample T test (*P*t&v)

*There were significant differences on the same variable in both the training and validation groups

*†There was significant difference on the same variable only validation group

### Results of the CNN models and comparisons between models

**Fig. 3:**
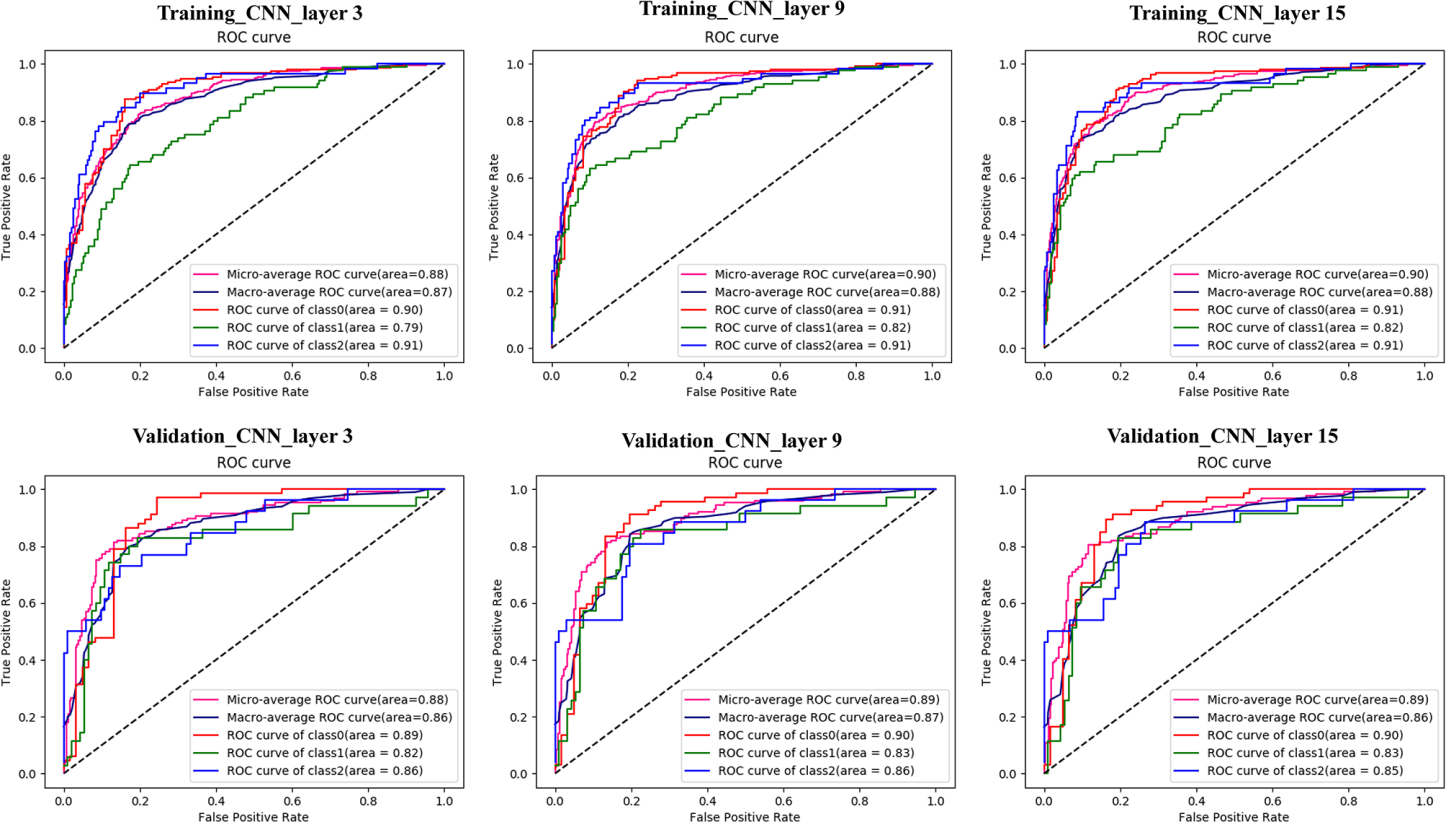
ROC curves of the CNN models. ROC, receiver operating characteristic; CNN, convolutional neural network; class 0/1/2 = very low and low/ intermediate/high risk groups

**Fig. 4:**
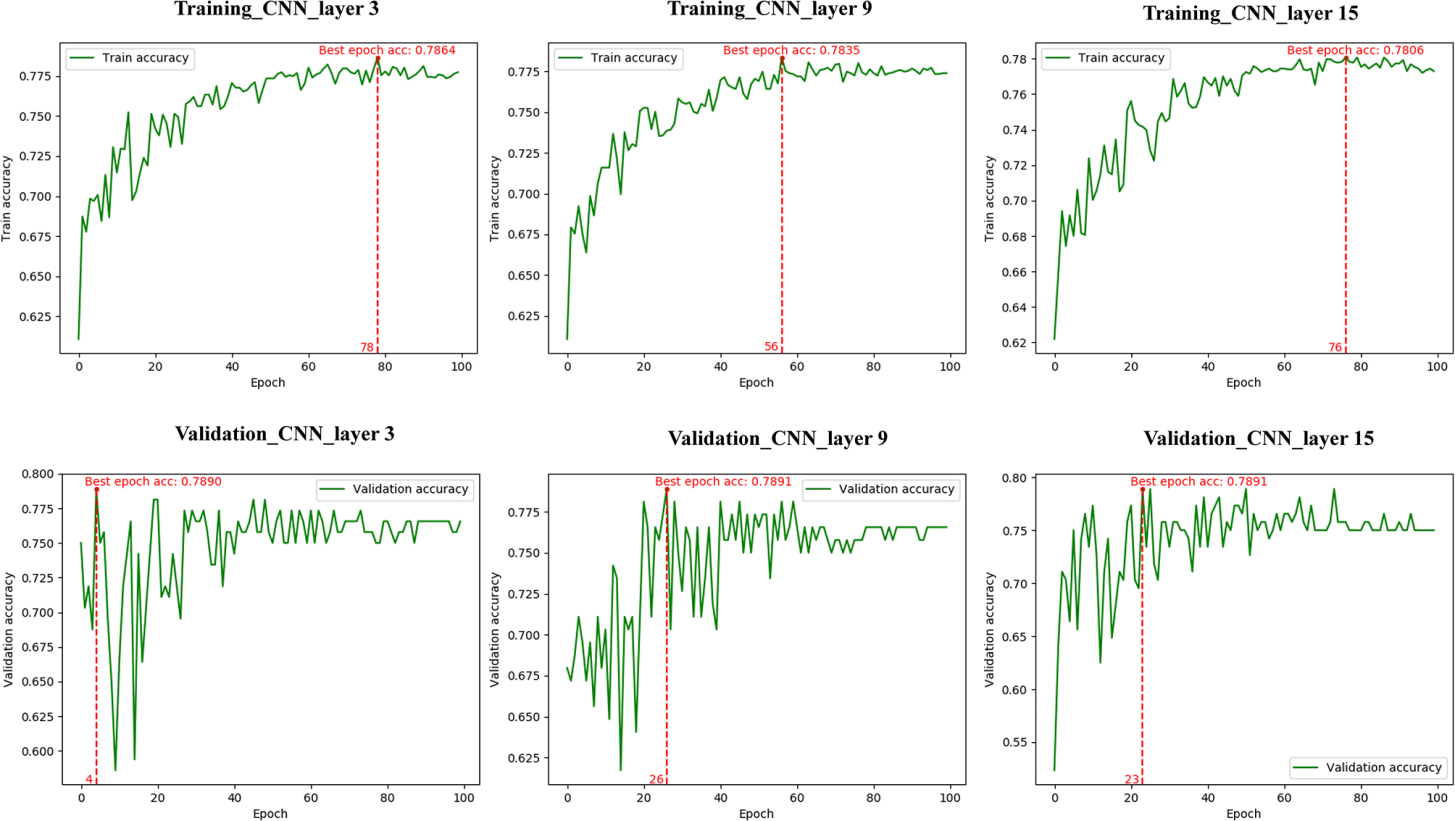
The best epoch acc for different CNN models. p 0/1/2 = possibility of very low and low/ intermediate/high risk

The AUROCs, sensitivity, specificity, TP, and FN are shown in Table 2; Fig. 3. Moreover, Table 3, and Figs. 3 and 4 demonstrated the best epoch accuracy (acc), kappa coefficient, micro average roc, macro average roc, ROC curve of class very low/low, ROC curve of class intermediate, and ROC curve class high risk groups in the training and validation dataset.

**Table 2:** Predictive performance of CNN in both training and validation cohort

	CNN_layer3			CNN_layer9			CNN_layer15		
Training cohort(297)	0 (154)	1 (84)	2 (59)	0 (154)	1 (84)	2 (59)	0 (154)	1 (84)	2 (59)
Predicted number	148	34	8	146	50	35	137	56	34
AUC	0.90	0.79	0.91	0.91	0.82	0.91	0.91	0.82	0.91
Sensitivity	0.961	0.4047	0.5253	0.948	0.5952	0.5953	0.8896	0.6667	0.5762
Specificity	0.6293	0.9014	0.9579	0.7552	0.9061	0.9537	0.8041	0.8450	0.9015
True positive	0.9610	0.4047	0.5254	0.9480	0.5952	0.5953	0.8896	0.6667	0.5762
False negative	0.0390	0.6953	0.4746	0.0520	0.4048	0.4047	0.1104	0.3333	0.4238
Validation cohort(128)	0 (67)	1 (35)	2 (26)	0 (67)	1 (35)	2 (26)	0 (67)	1 (35)	2 (26)
Predicted number	66	24	11	63	25	13	61	28	12
AUC	0.89	0.82	0.86	0.90	0.83	0.86	0.90	0.83	0.85
Sensitivity	0.985	0.6857	0.423	0.9402	0.7142	0.50	0.9104	0.8	0.4615
Specificity	0.6885	0.9139	1.0	0.7704	0.8817	0.9803	0.8360	0.8279	0.99
True positive	0.9850	0.6857	0.4230	0.9402	0.7142	0.5000	0.9104	0.800	0.4615
False negative	0.0150	0.3143	0.5770	0.0598	0.2858	0.5000	0.0896	0.2000	0.5385

Note.—Except where indicated, data in parentheses are numbers of tumors

**Table 3:** Diagnostic performance of CNN in risk tri-rating of GSTs in both training and validation cohorts

	Best epoch Acc	Kappa coefficient	Micro average roc	Macro average roc	Roc curve of class 0	Roc curve of class 1	Roc curve of class 2
Training cohort (297)							
CNN_layer3	0.7864	0.6512	0.88	0.87	0.90	0.79	0.91
CNN_layer9	0.7835	0.7114	0.90	0.88	0.91	0.82	0.91
CNN_layer15	0.7806	0.7171	0.90	0.88	0.91	0.82	0.91
Validation cohort (128)							
CNN_layer3	0.7891	0.6398	0.88	0.86	0.89	0.82	0.86
CNN_layer9	0.7891	0.6902	0.89	0.87	0.90	0.83	0.86
CNN_layer15	0.7891	0.7211	0.89	0.86	0.90	0.83	0.85

**Table 4:** The obuchowski index results of the three models

Cohort	Model 1	Model 2	Obuchowski index of Model 1	Obuchowski index of Model 2	*P*-value
Training cohort	CNN_layer3	CNN_layer9	0.8846467	0.9004745	**0.001187**
	CNN_layer3	CNN_layer15	0.8846467	0.9008081	**0.006965**
	CNN_layer9	CNN_layer15	0.9004745	0.9008081	0.878231
Validation cohort	CNN_layer3	CNN_layer9	0.8709226	0.8747248	0.644065
	CNN_layer3	CNN_layer15	0.8709226	0.8725235	0.861342
	CNN_layer9	CNN_layer15	0.8747248	0.8725235	0.583739

In the training dataset, CNN_layer9, and CNN_layer15 (Obuchowski index: 0.90 and 0.90, respectively) outperformed CNN_layer3 (Obuchowski index:0.88) in prediction of the three risk categories of gastric GISTs (*P* <.05), but no significant difference was found between these three models in the validation dataset (Table 4).

### Results of the probability distribution in the validation dataset

**Fig. 5:**
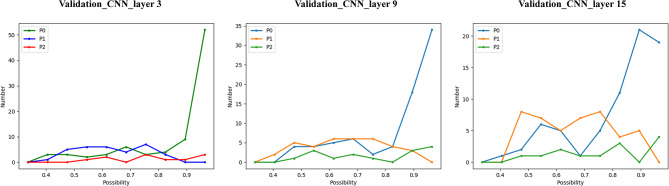
Line chart of probability distribution in validation data set

To assess the robustness of our CNN models, a line chart was plotted for each test image based on the probability of a tumor being classified as one of the three risk classifications in the validation dataset. Among these three risk classifications, the high-risk groups showed high probability when being diagnosed (all with probability > 0.51), which was higher than the very low/low and intermediate groups in these three CNN models (Fig. 5).

## Discussion

In this study, we present the results of a newly developed CNN model called EfficientNet_b1 that uses preoperative venous-phase CT images to predict the risk category of gastric GISTs. The findings of our study showed that CNN_layer 3/9/15 could accurately predict the risk classification of gastric GISTs in both the training dataset (all with AUROCs > 0.7) and validation dataset (all with AUROCs > 0.8), indicating that the CNN extracted suitable features for evaluating the risk in patients with gastric GISTs. To the best of our knowledge, this is the first study to report using a CNN applied to preoperative venous-phase CT images to predict the risk category of gastric GISTs, and the only study to compare the diagnostic efficacy of different CNN models obtaining upper and lower 3/9/15 layers of maximum tumor mask slice.

In China, the prognoses for GISTs are commonly stratified according to modified National Institute of Health (NIH) criteria, including size (2, 5, or 10 cm), mitotic index (< 5, 5–10, or > 10 mitoses per 50 HPFs), tumor site (gastric, small intestine, or other), and tumor rupture, because of their simplicity in clinical practice [19].Once GISTs have intermediate- or high-risk CT features, Surgery instead of endoscopy is the preferred treatment regardless of tumor size, and the difference in risk grade is closely related to the choice of surgical plan, surgical method and patient prognosis [20]. Therefore, accurate stratified risk assessment has important clinical reference value for the diagnosis, treatment and prognosis of patients [21]. The requirement for a precise risk rating has become a crucial task owing to emerging adjuvant systemic treatments. Recent guidelines state that only high-risk patients should be considered for adjuvant treatment, with the suggestion for intermediate-risk patients being ‘space for shared decision-making’ [22]. In the above­mentioned risk classification, high-risk GISTs are followed up by CT every 4–6 months, whereas GISTs with very low, low, or moderate risks are followed up by CT every 6–12 months [23]. Previous studies reported on the characteristic CT features of GISTs such as tumor size, calcification, ulcer, hemorrhage, intratumoral vessels, growth pattern, degree of enhancement, necrosis, and lymph node involvement, which may provide valuable information for predicting the risk rating of GISTs [7, 24–25]. However, the interpretations of CT findings were subjective and relied on radiologists. Our results found that tumor contour, growth pattern, necrosis, surface ulceration, LN involvement, hemorrhage, intratumoral vessel, peritumoral exudation, necrosis under the tumor wall, LD, SD, and LD/SD showed significant differences between the very low/low-risk, intermediate-risk, and high-risk groups in both the training dataset and validation dataset. However, it remains difficult for radiologists to predict the risk rating of gastric GISTs using these CT features because of their low occurrence rates and non-specificity.

The convolutional neural network (CNN), an advanced machine learning method, is a neural network able to learn complicated functions mapping an input to an output with no need for manually extracted characteristics [26–27]. In the field of gastrointestinal diseases, CNNs have begun to show promise for tumor detection, differential diagnosis, and risk assessment. Zhang et al. found that a CNN system based on endoscopic images showed better diagnostic performance in the detection of early gastric cancer than endoscopists with higher accuracy (85.1–91.2%) and stability [11]. Oh et al. and Liu et al. developed CNN systems using endoscopic ultrasound images that demonstrated higher diagnostic ability for GISTs than human assessments, including higher accuracy, sensitivity, and negative predictive value [27, 28]. A recent study reported that a deep learning machine for differentiating three risk levels of GISTs (high-risk, intermediate-risk, and low-risk GISTs) demonstrated an AUROC of 0.89 in the training dataset and 0.85 in the external validation dataset, showing better performance than a subjective model [14]. In this study, we developed CNN models using preoperative venous-phase CT images that achieved AUROCs above 0.7 for differentiating high-risk gastric GISTs from intermediate-risk and very low/low-risk gastric GISTs in the training dataset, and above 0.8 in the validation dataset. Furthermore, the diagnostic effect was obtained using the micro average roc and macro average roc, which are more credible because of the data imbalance in this multi-classification task. The micro average roc and macro average roc of the CNN models for differentiating the three risk categories of gastric GISTs were above 0.8 in both the training and validation datasets, showing high accuracy for the risk rating on venous-phase CT images. Previous studies using radiomics models confirmed that analyses using 3D or 2D-3D hybrid CNN models could supply more relevant information on lesions than 2D images, which may enhance the accuracy of discrimination [29–32]. In this study, we hypothesized that the diagnostic performance of CNN models could be affected by the tumor volume consists of different layers based on the maximum tumour mask slice, which can influence the accuracy of image segmentation. In the training dataset, the Obuchowski index was significantly higher with the CNN_layer9 and CNN_layer15 models than with the CNN_layer3 model (*P* <.05), providing preliminary evidence that more layers based on the maximum slice may improve the diagnostic performance of CNN models for predicting gastric GISTs. However, this difference was not confirmed in the validation dataset. Further research with an increased sample size is required to confirm the preliminary evidence. In our analysis, we showed detailed probability distributions for every subject in the validation dataset being classified as one of the three risk classifications, and these results manifested the high-risk groups showing high probability when being diagnosed (with all probability > 0.51), which were higher than those of the very low/low and intermediate groups for these three CNN models. These results also indicate the stability of the CNN models.

Our study is subject to several limitations. First, the numbers of patients in the intermediate and high-risk groups in both the training and validation datasets were lower than in the very low/low risk groups, and all venous-phase CT images were retrospectively obtained from one of only? four centers. As a result of the small number of included patients in intermediate and high-risk groups,

larger, multicentric trials are required to confirm these results. Second, a selective bias exits because the analysis was conducted retrospectively. Third, the tumor segmentation was finished manually, rather than being fully automated. The stability of our diagnostic model needs to be confirmed when using automatic segmentation. Finally, the venous-phase CT images were obtained from a variety of CT scanners, which may have resulted in potential confounding factors.

In conclusion, we developed and validated CNN models using preoperative venous-phase CT images to predict the risk categories of gastric GISTs with high accuracy and specificity, and these have potential for assisting clinical work in the imaging diagnosis of gastric GISTs. Although the volume of the lesions in the CNN_layer3/9/15 models are different, there is no difference in the identification of the risk category of gastric GISTs.

## Data Availability

The datasets generated and analyzed in the present study are not publicly available because the datasets will be further studied for publication in other articles, but are reasonably available from corresponding authors.

## Abbreviations

CNN: Convolutional neural network
GISTs: Gastrointestinal stromal tumors
AUROCs: Area under the receiver operating characteristic curves
NIH: National Institutes of Health
EUS: Endoscopic ultrasonography
CT_U_/CT_A_/CT_V_: CT value of unenhanced/arterial/venous phase
DEAP/DEPP: Degree of enhancement in arterial phase/portal venous phase
LD/SD: Longest dimension/short dimension
LN: Lymph node
TP/FN: True positive/false negative
AUC: Area under curve
ROC: Receiver operating characteristic
